# Colistin and Carbapenem-Resistant *Acinetobacter baumannii* Aci46 in Thailand: Genome Analysis and Antibiotic Resistance Profiling

**DOI:** 10.3390/antibiotics10091054

**Published:** 2021-08-30

**Authors:** Nalumon Thadtapong, Soraya Chaturongakul, Sunhapas Soodvilai, Padungsri Dubbs

**Affiliations:** 1 Department of Physiology, Faculty of Science, Mahidol University, Bangkok 10400, Thailand; nalumon.tha@mahidol.ac.th (N.T.); sunhapas.soo@mahidol.ac.th (S.S.); 2 Department of Microbiology, Faculty of Science, Mahidol University, Bangkok 10400, Thailand; soraya.cha@mahidol.ac.th; 3 Center of Microbial Genomics (CENMIG), Faculty of Science, Mahidol University, Bangkok 10400, Thailand

**Keywords:** *Acinetobacter baumannii*, colistin, carbapenems, multidrug resistant, WGS

## Abstract

Resistance to the last-line antibiotics against invasive Gram-negative bacterial infection is a rising concern in public health. Multidrug resistant (MDR) *Acinetobacter baumannii* Aci46 can resist colistin and carbapenems with a minimum inhibitory concentration of 512 µg/mL as determined by microdilution method and shows no zone of inhibition by disk diffusion method. These phenotypic characteristics prompted us to further investigate the genotypic characteristics of Aci46. Next generation sequencing was applied in this study to obtain whole genome data. We determined that Aci46 belongs to Pasture ST2 and is phylogenetically clustered with international clone (IC) II as the predominant strain in Thailand. Interestingly, Aci46 is identical to Oxford ST1962 that previously has never been isolated in Thailand. Two plasmids were identified (pAci46a and pAci46b), neither of which harbors any antibiotic resistance genes but pAci46a carries a conjugational system (type 4 secretion system or T4SS). Comparative genomics with other polymyxin and carbapenem-resistant *A. baumannii* strains (AC30 and R14) identified shared features such as CzcCBA, encoding a cobalt/zinc/cadmium efflux RND transporter, as well as a drug transporter with a possible role in colistin and/or carbapenem resistance in *A. baumannii*. Single nucleotide polymorphism (SNP) analyses against MDR ACICU strain showed three novel mutations i.e., Glu229Asp, Pro200Leu, and Ala138Thr, in the polymyxin resistance component, PmrB. Overall, this study focused on Aci46 whole genome data analysis, its correlation with antibiotic resistance phenotypes, and the presence of potential virulence associated factors.

## 1. Introduction

*Acinetobacter baumannii* is an opportunistic pathogenic bacterium that causes nosocomial infections in immunocompromised patients, especially patients treated in the intensive care unit (ICU) [1,2]. *A. baumannii* infections usually occur following: trauma, surgery, catheterization, or endotracheal intubation [3]. Moreover, this bacterium is well known for its multidrug resistant (MDR) characteristics, defined as resistance to at least one agent in three or more antibiotic categories [4], and as a nosocomial ESKAPE pathogen, a group including: *Enterococcus faecium*, *Staphylococcus aureus*, *Klebsiella pneumoniae*, *A. baumannii*, *Pseudomonas aeruginosa*, and *Enterobacter* species [5]. *A. baumannii* can resist almost all available antibiotics and it is possible for a strain to be pan drug resistant (PDR), which is defined as resistant to all agents in all antibiotic categories including last-resort antibiotics (carbapenems and polymyxins) [4].

Carbapenem-resistant *A. baumannii* or CRAB is considered by WHO (World Health Organization) as one of the leading threats to global human healthcare [6]. During the COVID-19 pandemic, CRAB infections have increased among COVID-19 patients who are subjected to long-term stays in the ICU [7,8]. The incidence of *A. baumannii* infection in Thailand is widely distributed in all regions of the country [9]. Clinical isolates of *A. baumannii* accounted for 15–16% of hospital-acquired bacteremia and CRAB comprises 70–88% of total *A. baumannii* clinical isolates [10]. Presence of the carbapenemase encoding genes, *blaOXA-23* or *blaOXA-51*, in combination with insertion sequence elements is frequently found in CRAB [11].

Unfortunately, colistin-resistant *A. baumannii* strains that are either MDR or PDR have been reported worldwide [12,13]. Polymyxins, including polymyxin B and colistin, are alternative last-line drugs used against CRAB [14]. Cationic polymyxin molecules target the polyanionic portions of the outer membrane of the bacterial envelope, specifically lipid A in lipopolysaccharide (LPS) [15]. Polymyxins bind lipid A and disrupt the outer membrane, causing cytoplasm leakage [16]. In Thailand, colistin-resistant *A. baumannii* isolates are found in 35–44% of pneumonia patients [17,18]. Among the colistin-resistant *A. baumannii* strains, four known mechanisms have been identified: (i) modification of lipid A, (ii) loss of LPS, (iii) disruption of outer membrane asymmetry, and (iv) efflux pumps [19]. Modification of lipid A involves increased addition of phosphoethanolamine (PEtN) to lipid A resulting from a mutation in the PmrAB two-component system. Increased expression of *pmrC*, which encodes lipid A phosphoethanolamine transferase, enhances addition of PEtN to either the 4′-phosphate or 1′-phosphate of lipid A [20]. Inactivation or complete loss of LPS occurs as a result of mutations in LPS biosynthesis genes such as: *lpxA*, *lpxC*, and *lpxD* [21]. Mutations in *vacJ*, encoding an outer membrane lipoprotein, and *pldA*, encoding an outer membrane phospholipase, result in accumulation of phospholipid, disrupting LPS organization, membrane asymmetry, and colistin binding [22]. Efflux pumps have also been shown to play important roles in the osmotic stress response and colistin resistance, specifically the AdeRS two-component system, which regulates expression of the AdeABC [23] and EmrAB efflux pumps [24].

Our previous report characterized MDR *A. baumannii* Aci46 that was isolated from a pus sample from a Thai patient at Ramathibodi hospital [25]. This strain was reported as a CRAB, i.e., resistant to imipenem. In the current study, we further explored the Aci46 resistance profile against other antibiotics including the alternative last-line drug, colistin. Since the genotypic characteristics of the MDR Aci46 were still unknown, genome studies were applied to identify antibiotic resistance genes, sequence type, and international clonal (IC) group of Aci46. In addition, the Aci46 genome was compared with other MDR *A. baumannii* and drug sensitive *A. baumannii* to characterize the unique gene features of the MDR (or CRAB) Aci46.

## 2. Results and Discussion

### 2.1. Antibiotic Resistance Phenotypes of Aci46

**Figure 1:**
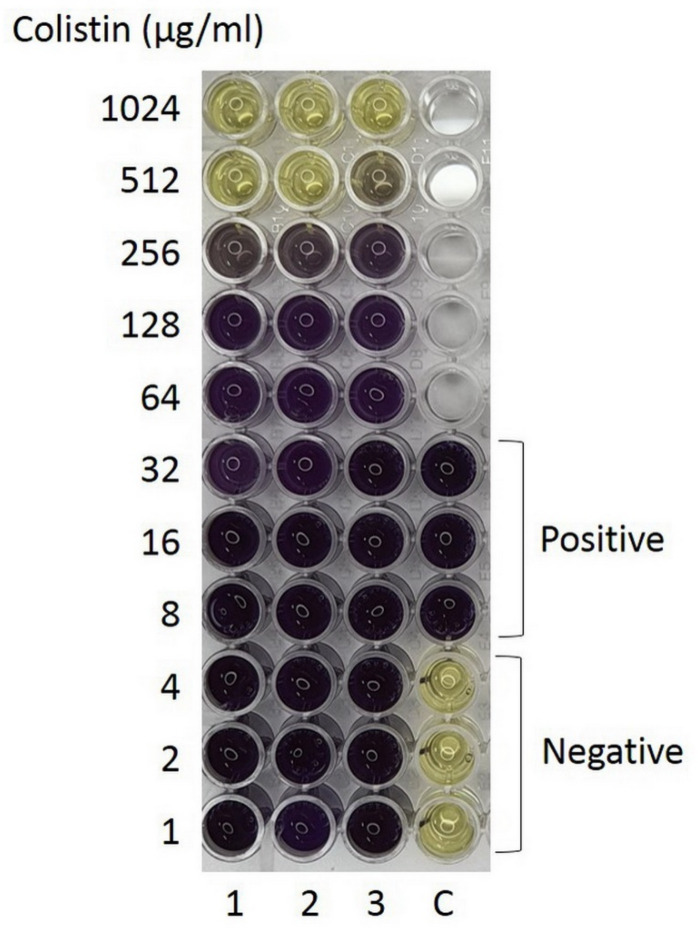
Minimum inhibitory concentration (MIC) determination by microdilution assay using MTT staining. Aci46 viable cells and positive control are shown in purple while dead cells or negative control are shown in yellow. The experiments were tested with three biological replicates.

A previous report has shown that Aci46 is susceptible to amikacin and resistant to cefoperazone-sulbactam, ceftazidime, ciprofloxacin, and imipenem [25]. To expand the antibiotic profile of Aci46, twenty drugs from eight classes (i.e., aminoglycosides, beta-lactams (and beta-lactam combined), carbapenems, quinolones, folate pathway blocks, phenicol, tetracycline, and colistin) were used in disk diffusion and microdilution assays. We found that Aci46 was resistant to all twenty drugs (Table 1, Figure S1 and Supplement Table S1) with a minimum inhibitory concentration (MIC) for colistin of 512 µg/mL (Figure 1). We have demonstrated that Aci46 is an XDR (extensively drug resistant) strain according to the definition described by Magiorakos et al. (non-susceptible to at least one agent in all but two antimicrobial categories specified for *Acinetobacter* spp.) [4].

### 2.2. Whole Genome Sequencing Data

To further investigate the genetic makeup of XDR Aci46, the chromosome and plasmids of Aci46 were subjected to next-generation sequencing. The summarized genome data is shown in Table 2. The Aci46 genome size is 3,887,827 bp with a GC content of 38.87%. The number of predicted protein coding sequences, rRNA genes, and tRNA genes were 3754, 3, and 63, respectively. We also identified two plasmids from the whole genome data, namely pAci46a and pAci46b. The size of pAci46a was 70,873 bp with a GC content of 33.39% while pAci46b was 8808 bp with a GC content of 34.31%. The number of predicted protein coding sequences for pAci46a and pAci46b were 102 and 11, respectively. No rRNA or tRNA genes were present in either case.

**Figure 2:**
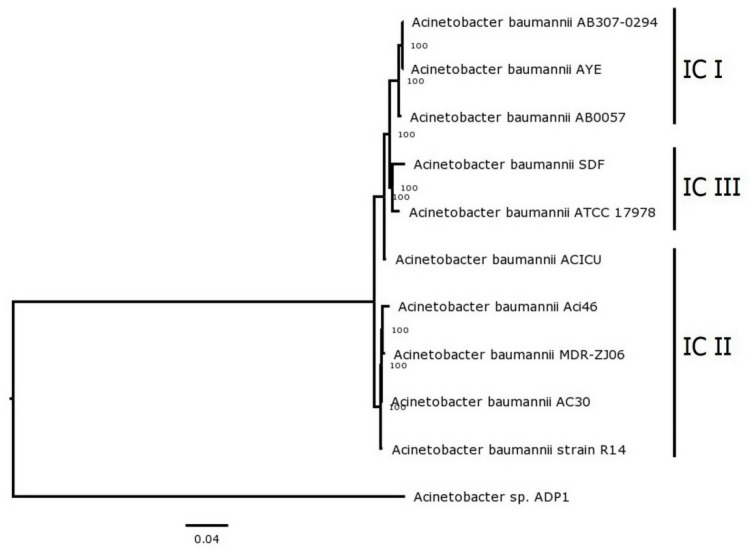
Phylogenetic analysis of *A. baumannii* Aci46 and ten sequences of *A. baumannii* genomes for identification of international clonal group (IC). The phylogenetic tree was constructed by RAxML version 8.2.11 using 100 single-copy genes with bootstrap values set to 100 replicates. Selected strains belonging to IC I, IC II, and IC III are labeled. The accession numbers of the strains are as follows: AYE (CU459141.1), AB0057 (CP001182.2), AB307-0294 (CP001172), AC30 (ALXD00000000), ACICU (CP031380.1), MDR-ZJ06 (CP001937.2), R14 (PUDB01000000), ATCC17978 (CP000521.1), SDF (CU468230.2), and *A. baylyi* ADP1 (CR543861).

The microbial taxonomy of Aci46 was confirmed as *A. baumannii* at 100% identity based on variation of 54 genes encoding ribosomal protein subunits. Typing of Aci46 was classified by multi-locus sequence typing (MLST) using Oxford and Pasture schemes. The sequence type (ST) of Aci46 was ST1962 (*gltA*-1, *gyrB*-3, *gdhB*-189, *recA*-2, *cpn60*-2, *gpi*-140, *rpoD*-3) based on the Oxford scheme [26], while it belonged to ST2 (*cpn60*-2, *fusA*-2, *gltA*-2, *pyrG*-2, *recA*-2, *rplB*-2, *rpoB*-2) based on the Pasture scheme [27]. ST2 based on Pasture scheme is a predominant ST of CRAB found in Thailand and Southeast Asia [10,28]. However, ST1962 based on the Oxford scheme has never been reported in Thailand. ST1962 has been reported in the USA for only one strain (PubMLST database, to be published). From our previous report, we knew that Aci46 harbored class 1 integrase [25]. However, the class 1 integron can be transferred across two *A. baumannii* IC groups, IC I and IC II [29]. Phylogenetic analysis of the Aci46 genome compared with ten genomes of *A. baumannii* from three different IC’s, with the *A. baylyi* ADP1 genome as an outgroup to root the tree, revealed that Aci46 is more closely related to *A. baumannii* MDR-ZJ06 and belongs to IC II (Figure 2).

### 2.3. Antibiotic Resistance Gene, Efflux Pump, and Virulence Gene Predictions

Based on the phenotypic characteristics of the antibiotic resistance profile, a search for the presence of antibiotic resistance genes and genes encoding efflux pumps associated with the XDR phenotype in Aci46 was undertaken. ResFinder, CARD, and NDARO databases were used to predict the antibiotic resistance genes and efflux pumps present in Aci46. We found that Aci46 harbored sixteen resistance genes against eight classes of drugs (i.e., aminoglycosides, beta-lactams/carbapenems, beta-lactams/cephalosporins, colistin, fluoroquinolones, macrolides, tetracycline, and sulfonamide) and twenty-two genes belonging to five classes of drug transporters (i.e., RND (resistance-nodulation-division) efflux systems, MFS (major facilitator superfamily) family transporter, ABC (ATP-binding cassette) transporter, MATE (multidrug and toxic compound extrusion) family transporter, and SMR (small multidrug resistance)) (Table 3). The genes, *blaOXA-23*, *blaOXA-66* or *blaOXA-51*-like, and *oprD* genes are present in Aci46 and they are known confer carbapenem-resistance [11]. These data correlate with presence of *blaOXA-23* and *blaOXA-51* in other CRAB isolates found in Thailand [30]. Moreover, class 1 and class 2 integrase genes and *blaOXA-23* are often found in XDR *A. baumannii* [31,32].

With regard to colistin resistance, we identified *lpxA* and *lpxC* in Aci46, these genes might play a role in loss of LPS and leading to colistin resistance [21]. In addition, we found four genes encoding efflux pumps (i.e., *adeR*, *adeS*, *emrA*, and *emrB*) and two genes with roles in lipid modification (i.e., *pmrA* and *pmrB*). AdeRS is a two-component system that regulates the expression of the AdeABC efflux pump, which is an RND efflux system [23]. The EmrAB efflux system belongs to the MFS family of transporters [24]. The PmrAB two-component system regulates PmrC expression. PmrC adds PEtN to lipid A [33]. In summary, the genotypic characteristics of Aci46 suggest that Aci46 resists colistin by way of lipid A modification, LPS loss, and AdeABC-mediated efflux.

Several virulence factors of *A. baumannii* have been identified by genome-based analysis [34]. The outer membrane protein, OmpA, which functions as a porin, is a key factor in virulence where it plays particular roles in cell invasion, development of cytotoxicity, and apoptosis [35]. Capsular polysaccharides and LPS are also virulence factors and contribute to serum resistance, biofilm formation, and escape from the host immune response [36]. *A. baumannii* uses combined strategies, namely, bacterial fitness and pathogenicity, to cause disease in humans [35]. PAI (pathogenicity islands), such as prophages and secretion systems, have also been implicated in virulence and pathogenicity [37,38]. In Aci46, we have identified pathogenicity islands comprising four prophages, one T4SS (type four secretion system), one T6SS (type six secretion system), and one ICE (integrative and conjugation element) (Table 4 and Supplement Table S2). No antibiotic resistance genes were found on the plasmids and prophages. Genotypic characteristics underlying the antibiotic resistance profile of Aci46 are found on its chromosome, and not on plasmids or other mobile genetic elements. T4SS is located in plasmid pAci46a, similar to pAC30c in *A. baumannii* AC30 and pAC29b in *A. baumannii* AC29 [2]. Generally, T4SS plays a role in the transfer antibiotic resistance genes via horizontal gene transfer [38]. Based on comparative genome analysis, T4SS loci are found in clinical isolates associated with hospital outbreaks [39]. The function of T4SS is still unclear in *A. baumannii*, but it might be implicated in pathogenicity or host–pathogen interaction [38,40]. Thus, pAci46a might play a role in pathogenesis instead of drug resistance. Moreover, we found *attL* (gtaataacaaagcaatcccgcagggttgcgacaaatagccctctaaatcgctctaattgcccctagattcaatttta) and *attR* (gtaataacaaagcaatcccgcagggttgcgacaaatagccctctaaatcgctctaattgcccctagattcaatttta) sites on pAci46a (or ICE region). It is possible that pAci46a could be a conjugative plasmid responsible for plasmid mobilization, similar to pAC30c and pAC29b [2]. T6SS injects toxic effectors into other bacteria in the same niche; therefore, it is an important factor for competitive killing and host colonization [34]. The plasmid, pAci46b, carries eleven genes encoding: one outer membrane receptor protein, one replication protein, and nine hypothetical proteins. The functions of pAci46b are still unclear.

### 2.4. Comparative Pangenomic Analysis against Other A. baumannii Strains

**Figure 3:**
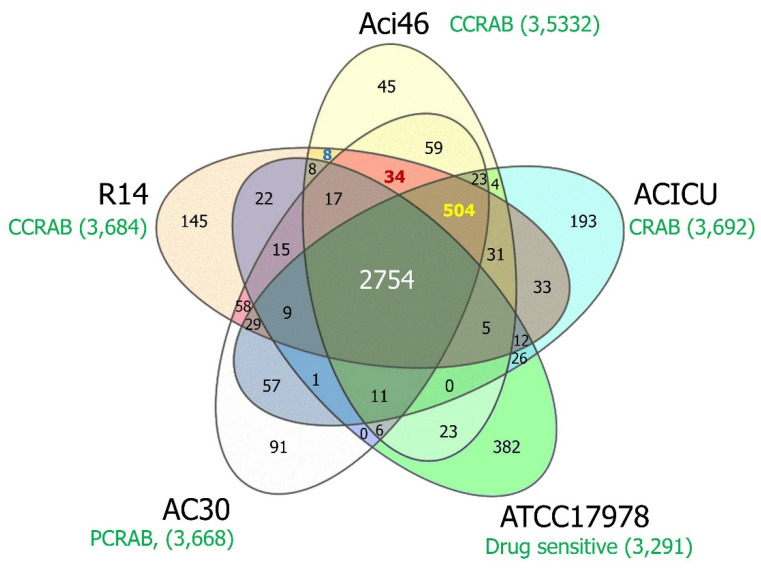
Venn diagram of comparative pangenome analysis among *A. baumannii* strains. The yellow, blue, green, white, and orange represent Aci46, ACICU, ATCC17978, AC30, and R14, respectively, with the number of total genes and the character of drug resistance in each genome (shown in green). The overlapped areas show genes encoding shared protein families among strains. The number of genes in core genes (Aci46, ACICU, ATCC17978, AC30, and R14), MDR (Aci46, ACICU, AC30, and R14), PCRAB (Aci46, AC30, and R14), and CCRAB (Aci46 and R14) the specific groups are labeled in white, yellow, red, and blue, respectively. CRAB: carbapenem-resistant *A. baumannii*, CCRAB: colistin and carbapenem-resistant *A. baumannii*, PCRAB: polymyxins and carbapenem-resistant *A. baumannii*.

In order to further explore the novel gene(s) that might be involved in the colistin and carbapenem-resistant phenotypes in Aci46, pangenome analysis was performed (Figure 3). Pangenome (all genes from all genomes) and core genes (present in all genomes) (Supplement Table S7) comprise 4605 and 2754 genes, respectively. We found that the number of strain-specific genes for Aci46, ACICU, ATCC17978, AC30, and R14 are 45, 193, 382, 91, and 145, respectively (Supplement Table S3). MDR (Aci46, ACICU, AC30, and R14) (Supplement Table S4), polymyxin and carbapenem resistance (renamed PCRAB) (Aci46, AC30, and R14), and colistin and carbapenem resistance (renamed CCRAB) (Aci46 and R14) groups contained 504, 34, and 8 accessory genes (i.e., genes present in specific genomes), respectively. We hypothesized that the accessory genes in PCRAB and/or CCRAB might be involved in resistance to polymyxins (polymyxin B and colistin). The thirty-four PCRAB specific genes encode: amidase, carbapenemase BlaOXA-23, CzcCBA cobalt/zinc/cadmium efflux RND transporter, twenty-three hypothetical proteins, lysophospholipid, nickel-cobalt-cadmium resistance protein, oxidoreductase, transcriptional regulator, and DNA-methyltransferase subunit M (Supplement Table S5). The eight CCRAB specific genes encode: one primosomal protein I and seven hypothetical proteins (Supplement Table S6). One of the PCRAB specific genes, *blaOXA-23*, is frequently reported in CRAB [41,42]. Interestingly, three genes (*czcA*, *czcB*, and *czcC*) encode CzcCBA cobalt/zinc/cadmium efflux RND transporters in PCRAB specific genes. CzcCBA cobalt/zinc/cadmium efflux RND transporters have functions in exporting cations (Co^2+^, Zn^2+^, and Cd^2+^) from the cytoplasm and confer heavy metal resistance [43,44], and are reported to be associated with the XDR phenotype in *A. baumannii* [45]. This efflux system might function in colistin resistance in PCRAB, including Aci46, by exporting colistin and polymyxin (cationic molecules). In the case of CCRAB specific genes, their functions are unknown.

### 2.5. Pairwise SNP Analysis

**Figure 4:**
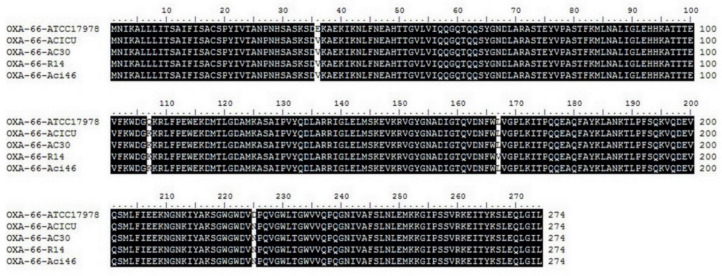
Comparisons of deduced amino acid sequences of *blaOXA-66* from five strains of *A. baumannii* by multiple sequence alignment.

Although we identified genes that were specific to PCRAB that encoded CzcCBA efflux pumps, known MDR genes were among the core genes (Supplement Table S7). Thus, in order to identify resistance-associated mutations in MDR and CCRAB strains, non-synonymous SNPs between Aci46 and ATCC17978 and between Aci46 and ACICU were identified. SNPs within known MDR genes from the core genes are listed in Table 5. Twenty genes show SNPs in Aci46 vs. ATCC17978 i.e., seven antibiotic resistance genes (*blaADC-25*, *blaOXA-66*, *lpxA*, *lpxC*, *pmrB*, *gyrA*, and *gyrB*) and thirteen drug transporter genes (*adeA*, *adeB*, *adeF*, *adeG*, *adeH*, *adeJ*, *adeR*, *adeS*, *opmH*, *emrB*, *mdfA*, *macB*, and *abeS*). The deduced amino acid sequences of these genes among the MDR strains were similar. For example, the deduced amino acid sequences of *blaOXA-66* or *blaOXA-51*-like genes in Aci46, ACICU, AC30, and R14 showed conserved amino acids at Val36, Lys107, and Asn225 while ATCC17978 contained Glu36, Gln107, and Asp225 (Figure 4). In 2015, the Trp22Met mutation of *blaOXA-51* was linked with carbapenem resistance function in *A. baumannii* [46]. This result suggested that amino acid sequences of antibiotic resistant and drug transporter proteins in MDR strains could be different from drug sensitive strains and might be linked to drug resistance level. For colistin resistance, we found three genes (*blaADC-25*, *pmrB*, and *gyrB*) that were mutated in Aci46 vs. ACICU. Of these only *pmrB* is related to colistin resistance. From comparisons of Aci46 vs. ACICU and Aci46 vs. ATCC17978, mutations in PmrB were detected in three positions: Ala138Thr, Pro200Leu, and Glu229Asp (Table 5). Known PmrB mutations that confer colistin resistance are Leu9-Gly12 deletion, Ala22Val, Ile232Thr, and Gln270Pro [47,48]. Hence, Ala138Thr, Pro200Leu, and Glu229Asp mutations in Aci46 PmrB might be novel mutations involved in colistin resistance.

## 3. Materials and Methods

### 3.1. Bacterial Strains

*A. baumannii* Aci46 was isolated from a male Thai patient treated at Ramathibodi hospital, Thailand [25]. This strain was isolated from a pus sample, identified by routine biochemical test, and confirmed by *blaOXA51*-like gene detection [25,49]. Aci46 was cultured on MHA (Mueller Hinton Agar, BD Difco, Eysins, Switzerland) and incubated at 37 °C for overnight.

### 3.2. Antibiotic Susceptibility Testing by Disk Diffusion and Microdilution

Antibiotic susceptibility was determined by disk diffusion method for 19 drugs (gentamicin, kanamycin, streptomycin, cephalothin, cefoxitin, cefotaxime, ceftazidime, ceftriaxone, ampicillin-clavulanic acid, imipenem, meropenem, ciprofloxacin, nalidixic acid, norfloxacin, trimethoprim, trimethoprim-sulfamethoxazole, ampicillin, chloramphenicol, and tetracycline) (Oxoid, Thermo Fisher Scientific, Waltham, MA, USA) and microdilution method for colistin. Aci46 was streaked on MHA and incubated overnight. Colonies were picked and resuspended in normal saline solution at 1 × 10^8^ cfu/mL (OD_600_ = 0.08–0.12 or 0.5 McFarland). The cell suspension was spread on MHA using a cotton swab. Antibiotic disks were placed on the agar surface. After incubation at 37 °C for 20–24 h., the zones of inhibition were measured and the results were interpreted following the CLSI (Clinical and Laboratory Standard Institute) guideline [50]. For the microdilution method, cell suspensions of Aci46 were diluted in CAMHB (Cation-Adjusted Mueller Hinton Broth, BD Difco, Eysins, Switzerland) to 1 × 10^6^ cfu/mL. Two-fold serial dilutions of colistin were prepared (1–1024 µg/mL) and mixed with 5 × 10^5^ cfu/mL of Aci46 in 200 µL total volume. After incubation at 37 °C for 20–24 h., the minimum inhibitory concentration was observed and cell viability was measured by MTT-based staining [51]. Ten µL of 5 mg/mL MTT (3-(4,5-dimethylthiazol-2-yl)-2,5-diphenyl-tetrazolium bromide (Invitrogen, Life Technologies, Carlsbad, CA, USA) in phosphate buffer saline was added in 100 µL of cell culture and incubated at 37 °C, 200 rpm, 1 h in the dark. One hundred µL of 10% SDS (sodium dodecyl sulfate, Merck, Darmstadt, Germany) and 50% DMSO (dimethyl sulfoxide, Sigma-Aldrich, St. Louis, MO, USA) was added and continually incubated at 37 °C, 200 rpm, 2 h in the dark. The absorbance of formazan dissolution was detected at 570 nm using a microplate reader (Azure Ao Absorbance Microplate Reader, Azure Biosystems, Dublin, CA, USA). Relative optical density at 570 nm was calculated by dividing OD_570_ of drug-containing wells with the OD_570_ of drug-free wells [52]. The cut-off for no detection was the relative OD_570_ of 0.1. Viable cells under MTT staining can also be observed by the naked eye i.e., color change from yellow to purple. Colistin resistance was determined by CLSI guideline (MIC ≥ 4 µg/mL; resistant) [50]. *Escherichia coli* ATCC25922 was selected to be a control strain for disk diffusion and microdilution methods.

### 3.3. Genomic DNA Extraction and Whole Genome Sequencing

Whole genomic DNA of Aci46 was extracted using a modified Marmur procedure [53]. Briefly, Aci46 cells were harvested from 3 mL of cell culture in CAMHB and resuspended in EDTA-saline (0.01 M EDTA and 0.15 M NaCl, pH 8.0). Thirty µL of 110 mg/mL lysozyme and 10 µL of 20 mg/mL RNase A were added and incubated at 37 °C for 2 h. After incubation, 80 µL of 20% SDS and 10 µL of 5 mg/mL proteinase K were added and incubated again at 65 °C, 30 min. Then, 5 M NaCl was added at 0.5 volume followed by phenol-chloroform extraction. The upper liquid phase was transferred to a new 1.5 mL microcentrifuge tube. A 0.25 volume of 5 M NaCl and a 0.1 volume of 3 M sodium acetate were added and mixed well. Ice-cold absolute ethanol was added at 2 volumes and inverted gently. The DNA pellet was hooked and transferred into a new 1.5 mL microcentrifuge tube, air dried, and resuspended in DNase-RNase-free water. Quality and quantity of DNA were measured by UV spectrophotometer (OD_260_/OD_280_ and OD_260_/OD_230_ ratio) (DeNovix DS-11 FX+ spectrophotometer, DeNovix, Wilmington, DE, USA), Qubit dsDNA BR assay kit (Invitrogen, Life Technologies, Carlsbad, CA, USA), and 1% agarose gel electrophoresis (Bio-rad, Hercules, CA, USA). One hundred ng of extracted DNA was used for library preparation using TruSeq Nano DNA Kit (Illumina, San Diego, CA, USA) followed by pair-end sequencing on Illumina HiSeq platform (Illumina, San Diego, CA, USA).

### 3.4. Genome Assembly, Annotation, and Pathogenicity Island Prediction

Raw sequence data of Aci46 were trimmed by Trim Galore version 0.6.3 [54] and the quality was checked using FastQC version 0.11.8 [55]. Trimmed reads were assembled using SPAdes version 3.12.0 [56], corrected assembly error by Pilon version 1.23 [57], and calculated genome coverage by SAMTools version 1.3 [58] in PATRIC (Pathosystems Resource Integration Center) version 3.6.9 [59]. The quality of de novo assembled contigs was assessed by QUAST version 5.0.2 [60] and visualized using Bandage version 0.8.1 [61]. Coding sequences and functional genes were annotated using RASTtk (Rapid Annotation using Subsystem Technology toolkit) [62]. Antibiotic resistance genes were predicted using ResFinder version 4.1 [63], CARD (Comprehensive Antibiotic Resistance Database) [64], and NDARO (National Database of Antibiotic Resistant Organisms) [65] databases. Pathogenicity islands (type 4 secretion system and type 6 secretion system) and prophages were predicted using VRprofile version 2.0 [66] and PHASTER (PHAge Search Tool Enhanced Release) [67], respectively. In plasmid analysis, trimmed reads were used for searching and assembling plasmid sequences using plasmidSPAdes version 3.12.0 [68] in PATRIC version 3.6.9 server [59]. Quality control, annotation, and pathogenicity island predictions of plasmids were assessed using the same tools as with genomic analysis.

### 3.5. MLST and Phylogenetic Analysis

Identification of *A. baumannii* Aci46 was confirmed by rMLST (ribosomal multilocus sequence typing) [69] in PubMLST server [70]. The ST (sequence typing) of Aci46 was identified by MLST (multilocus sequence typing) according to the Pasture scheme (based on seven housekeeping genes *cpn60*, *gdhB*, *gltA*, *gpi*, *gyrB*, *recA*, and *rpoD*) [27] and Oxford scheme (based on seven housekeeping genes *cpn60*, *fusA*, *gltA*, *pyrG*, *recA*, *rplB*, and *rpoB*) [26] in PubMLST server [70]. The IC (international clonal) group of Aci46 was determined by phylogenetic relationship analysis using RAxML version 8.2.11 [71] based on 100 single-copy genes selected by PATRIC PGFams program [72] in PATRIC version 3.6.9 server [59]. The phylogenetic tree was visualized by FigTree [73]. Genomes of *A. baumannii* from IC I, IC II, IC III groups were used to identify the IC of Aci46. IC I group included *A. baumannii* strain AYE (CU459141.1) [74], AB0057 (CP001182.2) [75], and AB307-0294 (CP001172) [75]. IC II group included *A. baumannii* strain AC30 (ALXD00000000) [2], ACICU (CP031380.1) [76], MDR-ZJ06 (CP001937.2) [77], and R14 (PUDB01000000) [78]. IC III included *A. baumannii* strain ATCC17978 (CP000521.1) [79] and SDF (CU468230.2) [74]. The outgroup strain was *A. baylyi* ADP1 (CR543861) [80].

### 3.6. Comparative Pangenome and Pairwise SNP Analysis

Genomic data from five strains comprised of colistin and carbapenem-resistant Aci46, polymyxin B and carbapenem-resistant AC30 (ALXD00000000) [2], colistin and carbapenem-resistant R14 (PUDB01000000) [78], wild-type or drug sensitive ATCC17978 (CP000521.1) [79], and colistin-sensitive and carbapenem-resistant ACICU (CP031380.1) [76] were compared using the Protein Family Sorter with PATRIC genus-specific families (PLfams) strategy in PATRIC server [59]. In pairwise SNP analysis, genome data of Aci46 was aligned with the ATCC17978 genome (CP000521.1) and ACICU (CP031380.1) using BWA-mem aligner [81] and SNP calling by FreeBayes [82]. The deduced amino acid sequences of *blaOXA-66* from five genomes were compared by Clustal Omega in EMBL-EBI [83].

## 4. Conclusions

In summary, this study reported the genome data of colistin and carbapenem-resistant *A. baumannii* Aci46, which was isolated from a patient in a Thai hospital. The MLST genotype of Aci46 is Pasture ST2 which is a predominant ST found in Thailand and Oxford ST1962 which has never been reported in Thailand. The predicted antibiotic resistance genes (for example, *blaOXA-23*, *blaOXA-66*, and *blaADC-25*) are on the chromosome, not plasmids. Based on pangenome analysis, we found that the CzcCBA cobalt/zinc/cadmium efflux RND transporter might be involved in conferring resistance to colistin and/or carbapenem. From SNP analysis, we identified three points of non-synonymous mutations in *pmrB* (412G > A, 599C > T, and 687A > C) that change amino acid sequences. These amino acid changes, specifically Glu229Asp, Pro200Leu, and Ala138Thr may confer colistin resistance in MDR *A. baumannii* strains.

## Figures and Tables

**Table 1:** Antibiotic susceptibility profiling of *A. baumannii* Aci46 by disk diffusion method.

Class of Antibiotic	Drug	ID	Dose	Zone of Inhibition (mm) *	Interpretation
Aminoglycosides	Gentamicin	CN10	10 µg	0	R
	Kanamycin	K30	30 µg	0	R
	Streptomycin	S10	10 µg	0	R
Beta-lactams	Ampicillin	AMP10	10 µg	0	R
	Cephalothin	KF30	30 µg	0	R
	Cefoxitin	FOX30	30 µg	0	R
	Cefotaxime	CTX30	30 µg	0	R
	Ceftazidime	CAZ30	30 µg	0	R
	Ceftriaxone	CRO30	30 µg	0	R
Beta-lactam combined	Amoxycillin/clavulanic acid	AMC30	30 µg	0	R
Carbapenems	Imipenem	IPM10	10 µg	7–8	R
	Meropenem	MEM10	10 µg	0	R
Quinolones	Ciprofloxacin	CIP5	5 µg	0	R
	Nalidixic acid	NA30	30 µg	0	R
	Norfloxacin	NOR10	10 µg	0	R
Folate pathway blocks	Trimethoprim	W5	5 µg	0	R
	Trimethoprim-sulfamethoxazole	SXT25	25 µg	0	R
Phenical	Chloramphenicol	C30	30 µg	10–11	R
Tetracycline	Tetracycline	TE30	30 µg	0	R

* The ranges of inhibition zones were calculated from three individual replicates and “0” means no inhibition zone.

**Table 2:** Standard data of Aci46 genome features.

**Feature**	**Chromosome**	
Total number of bases (bp)	3,887,827	
G + C content (%)	38.87	
number of contigs	80	
genome coverage (x)	686.7	
number of coding sequences	3754	
rRNA	3	
tRNA	63	
N50	135,425	
L50	11	
**Feature**	**pAci46a**	**pAci46b**
Total number of bases (bp)	70,873	8808
G + C content (%)	33.39	34.31
number of contigs	1	1
genome coverage (x)	1189.0	7787.5
number of coding sequences	102	11
rRNA	0	0
tRNA	0	0

**Table 3:** List of predicted antibiotic resistance and drug transporter genes.

Target		Location				Product
	Gene	Contig	Start	Stop	Length (bp)	
**Antibiotics**						
Aminoglycosides	*aph(3″)-Ib*	Aci46-0022	7765	8568	804	Aminoglycoside 3*″*-phosphotransferase
	*aph(6)-Id*	Aci46-0022	6929	7765	837	Aminoglycoside 6-phosphotransferase
	*armA*	Aci46-0031	687	1460	774	hypothetical protein
Beta-lactams/ carbapenems	*blaOXA-23*	Aci46-0058	1667	2488	882	Class D beta-lactamase OXA-23
	*blaOXA-66*	Aci46-0018	65,582	66,406	825	Class D beta-lactamase OXA-66 or OXA-51-like
	OprD family	Aci46-0035	24,815	26,131	1317	Outer membrane low permeability porin, OprD family
Beta-lactams/ cephalosporins	*blaADC-25*	Aci46-0017	86	1237	1152	Class C beta-lactamase ADC-25
Colistin	*lpxA*	Aci46-0001	58,724	59,512	789	Acyl-[acyl-carrier-protein]--UDP-N-acetylglucosamine O-acyltransferase
	*lpxC*	Aci46-0006	139,160	140,062	903	UDP-3-O-[3-hydroxymyristoyl] N-acetylglucosamine deacetylase
	*pmrA*	Aci46-0016	34,812	35,486	675	Polymyxin resistant component PmrA
	*pmrB*	Aci46-0016	35,512	36,846	1335	Polymyxin resistant component PmrB
Fluoroquinolones	*gyrA*	Aci46-0003	54,354	57,068	2715	DNA gyrase subunit A
	*gyrB*	Aci46-0028	32,254	34,722	2469	DNA gyrase subunit B
Macrolide	*mph(E)*	Aci46-0054	2308	3192	885	Mph(E) family macrolide 2′-phosphotransferase
	*msr(E)*	Aci46-0054	3248	4723	1476	ABC-F type ribosomal protection protein Msr(E)
Tetracycline	*tetA*	Aci46-0022	2357	3556	1200	Tetracycline resistance protein
	*tetR*	Aci46-0022	3638	4261	624	Tetracycline resistance regulatory protein TetR
Sulfonamide	*sul2*	Aci46-0064	97	912	816	Dihydropteroate synthase type-2
**Drug transporters**						
RND efflux system	*adeA*	Aci46-0019	60,468	61,658	1191	RND efflux system, membrane fusion protein
	*adeB*	Aci46-0019	61,655	64,765	3111	RND efflux system, inner membrane transporter
	*adeF*	Aci46-0011	61,731	62,951	1221	RND efflux system, membrane fusion protein
	*adeG*	Aci46-0011	62,958	66,137	3180	RND efflux system, inner membrane transporter
	*adeH*	Aci46-0011	6389	6835	447	Efflux transport system, outer membrane factor (OMF) lipoprotein
	*adeI*	Aci46-0016	53,597	54,847	1251	RND efflux system, membrane fusion protein
	*adeJ*	Aci46-0016	50,408	53,584	3177	RND efflux system, inner membrane transporter
	*adeK*	Aci46-0016	48,954	50,408	1455	Efflux transport system, outer membrane factor (OMF) lipoprotein
	*adeN*	Aci46-0001	43,293	43,946	654	Transcriptional regulator, AcrR family
	*adeR*	Aci46-0019	59,579	60,322	744	Two-component transcriptional response regulator, LuxR family
	*adeS*	Aci46-0019	58,474	59,547	1074	Osmosensitive K+ channel histidine kinase KdpD
	*mexT*	Aci46-0029	34,598	35,587	990	Transcriptional regulator, LysR family
	*opmH*	Aci46-0022	48,920	50,266	1347	Outer membrane channel TolC (OpmH)
MFS family transporter	*emrA*	Aci46-0024	20,758	21,909	1152	Multidrug efflux system EmrAB-OMF, membrane fusion component EmrA
	*emrB*	Aci46-0024	19,228	20,751	1524	Multidrug efflux system EmrAB-OMF, inner-membrane proton/drug antiporter EmrB
	*mdfA*	Aci46-0002	123,994	125,223	1230	Multidrug efflux pump MdfA/Cmr (of MFS type), broad spectrum
ABC transporter	*macA*	Aci46-0012	77,649	78,989	1341	Macrolide-specific efflux protein MacA
	*macB*	Aci46-0012	78,992	80,986	1995	Macrolide export ATP-binding/permease protein MacB
MATE family transporter	*abeM*	Aci46-0007	67,926	69,272	1347	Multidrug efflux transporter MdtK/NorM (MATE family)
SMR	*abeS*	Aci46-0011	56,864	57,193	330	small multidrug resistance family (SMR) protein

**Table 4:** Predicted pathogenicity islands in WGS of *A. baumannii* Aci46.

Pathogenicity Island	Contig	Start	Stop	Length (bp)	GC Content (%)	Number of CDS
Prophage-1	Aci46-0033	9107	23,948	14,842	40.09	14
Prophage-2	Aci46-0033	27,330	37,053	9724	40.71	11
Prophage-3	Aci46-0036	785	30,936	30,152	38.52	46
Prophage-4	Aci46-0041	806	17,770	16,965	40.29	29
Type 4 secretion system, T4SS	pAci46a	20,944	48,690	27,747	34.60	32
Type 6 secretion system, T6SS	Aci46-0010	8673	27,581	19,412	36.92	15
Integrative and conjugation element, ICE	pAci46a	1	70,873	70,873	33.39	94

**Table 5:** List of non-synonymous SNPs in antibiotic resistance genes and efflux pumps from core gene group.

Target	Gene	Aci46 vs. ATCC17978		Aci46 vs. ACICU	
		Nucleotide Change	Amino Acid Change	Nucleotide Change	Amino Acid Change
**Antibiotic Resistance Genes**					
Beta-lactams/ cephalosporins	*blaADC-25*	356TG > AA	Val119Glu	238C > A	Arg80Ser
		448C > A	Gln150Lys	448C > A	Gln150Lys
		499C > T	Pro167Ser	487C > A	Gln163Lys
		739G > A	Gly247Ser	499C > T	Pro167Ser
		843AGGGTT > GGGTCG	GlnGlyPhe281GlnGlyArg	547A > G	Arg183Gly
		1022A > C	Asn341Thr	739G > A	Gly247Ser
				843AGGGTT > GGGTCG	GlnGlyPhe281GlnGlyArg
				932G > A	Ser311Asn
				1020CAA > TAC	ThrAsn340ThrThr
				1135G > A	Asp379Asn
Beta-lactams/ carbapenems	*blaOXA-66*	107A > T	Glu36Val		
		315CGGGC > TGGTA	AspGlyGln105AspGlyLys		
		673G > A	Asp225Asn		
Colistin	*lpxA*	391T > C	Tyr131His		
	*lpxC*	358T > C	Cys120Arg		
		859A > G	Asn287Asp		
	*pmrB*	412G > A	Ala138Thr	412G > A	Ala138Thr
		599C > T	Pro200Leu	599C > T	Glu229Asp
		687A > C	Glu229Asp	687A > C	Pro200Leu
		1331C > T	Ala444Val		
Fluoroquinolones	*gyrA*	173C > T	Ser58Leu		
	*gyrB*	2059A > G	Ile687Val	1738T > C	Tyr580His
**Drug Transporters**					
RND efflux system	*adeA*	70A > G	Lys24Glu		
	*adeB*	917G > T	Gly306Val		
		1279AAT > TCG	Asn427Ser		
		1654G > A	Ala552Thr		
		1928C > A	Ala643Asp		
		1936A > T	Thr646Ser		
		2191C > T	Leu731Phe		
	*adeF*	1163A > G	Asn388Ser		
	*adeG*	1540G > T	Val514Leu		
	*adeH*	214A > G	Thr72Ala		
		683T > G	Val228Gly		
	*adeJ*	2482A > G	Lys828Glu		
	*adeR*	358G > A	Val120Ile		
		407C > T	Ala136Val		
	*adeS*	515T > C	Leu172Pro		
		557G > T	Gly186Val		
		802A > C	Asn268His		
		908A > T	Tyr303Phe		
		1042G > A	Val348Ile		
	*opmH*	386A > G	Lys129Arg		
		512A > G	Asn171Ser		
MFS family transporter	*emrB*	715A > G	Ile239Val		
	*mdfA*	1157C > T	Ala386Val		
ABC transporter	*macB*	1462G > A	Val488Ile		
SMR	*abeS*	121A > G	Ile41Val		
		165G > C	Met55Ile		
		250G > T	Val84Leu		
		268CTTA > TTGG	LeuThr90LeuAla		
		292ATC > GTG	Ile98Val		

## Data Availability

The whole genome and plasmid sequences of *A. baumannii* Aci46 have been deposited at DDBJ/ENA/GenBank under the BioProject ID PRJNA739068.

## Supplementary Materials

Supplementary data are available online at https://www.mdpi.com/article/10.3390/antibiotics10091054/s1, Supplement Figure S1: The relative optical density at 570 nm for determination of minimum inhibitory concentration (MIC) by microdilution assay using MTT staining, Supplement Table S1: Guideline for interpretation of disk diffusion results, Supplement Table S2: Details of pathogenicity islands, Supplement Table S3: List of strain-specific genes, Supplement Table S4: List of multidrug resistant-specific genes (Aci46-ACICU-AC30-R14), Supplement Table S5: List of polymyxins and carbapenem-resistant-specific genes (Aci46-AC30-R14), Supplement Table S6: List of colistin and carbapenem-resistant-specific genes (Aci46-R14), Supplement Table S7: List of core genes (present in all genomes).
