# Supplementary material for: Colistin and Carbapenem-Resistant Acinetobacter baumannii Aci46 in Thailand: Genome Analysis and Antibiotic Resistance Profiling
Source: Antibiotics (Basel). 2021 Aug 30;10(9):1054. doi: 10.3390/antibiotics10091054 (PMC8468411; doi:10.3390/antibiotics10091054)
Supplement: Supplementary file 1 [file antibiotics-10-01054-s001.zip › NT2_suppl figure.pdf]

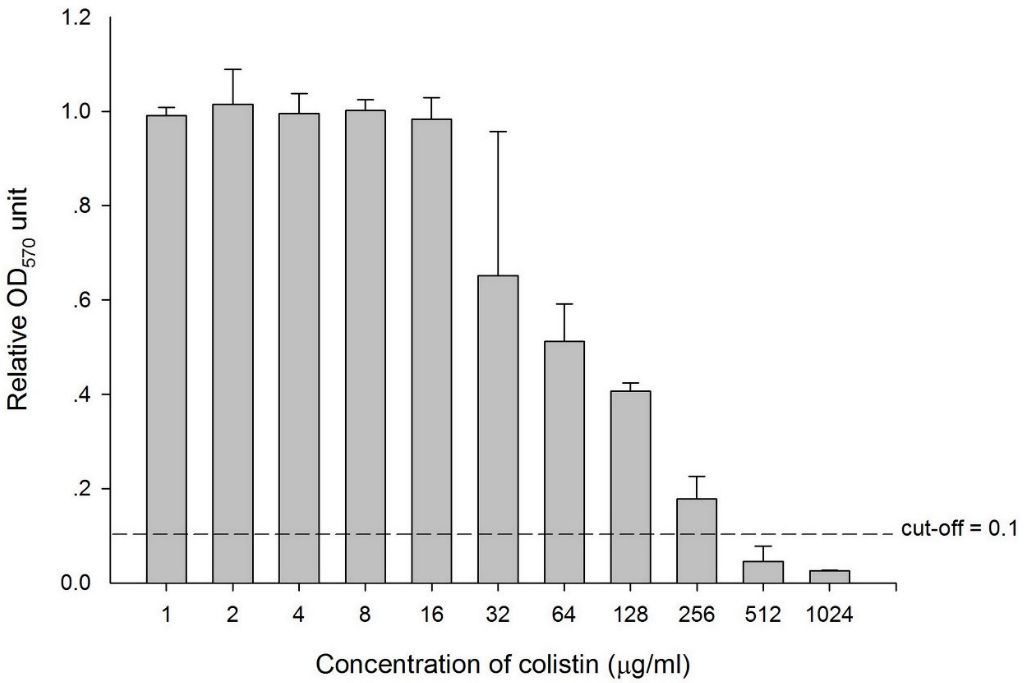

Supplement Figure S1. The relative optical density at 570 nm for determination of minimum inhibitory concentration (MIC) by microdilution assay using MTT staining. The results are mean values from three individual triplicate samples and error bars are standard deviation (SD). Cut-off for interpreting the detected cell alive is 0.1.
